# Incidental Findings in Computed Tomography Examination of the Head in Rabbits and Guinea Pigs

**DOI:** 10.3390/vetsci10080504

**Published:** 2023-08-04

**Authors:** Francesca Del Chicca, Caterina Puccinelli, Daniele Petrini, Simonetta Citi

**Affiliations:** 1 Clinic for Diagnostic Imaging, Vetsuisse Faculty, University of Zurich, Winterthurerstrasse 258c, 8057 Zurich, Switzerland; 2 Department of Veterinary Sciences, University of Pisa, Via Livornese, San Piero a Grado, 56122 Pisa, Italy

**Keywords:** computed tomography (CT), rabbit, guinea pig, head, incidental finding

## Abstract

**Simple Summary:**

Computed tomography (CT) is a commonly used imaging modality for the examination of the heads of rabbits and guinea pigs. This study reviewed 60 clinical records and CT examinations of the heads of rabbits and 65 of guinea pigs and investigated the incidental findings not directly related to the indications for the study and the suspected diagnoses. Most often, incidental findings involved the ears and the nose in both species. Mineralization in soft tissues is also often visible. The prevalence of incidental findings in CT examination of the analyzed species is high: 66.7% in rabbits and 64.6% in guinea pigs.

**Abstract:**

(1) Background: Rabbits and guinea pigs are popular pet animals and often undergo computed tomography (CT) examination for assessment of pathologies of the head. The goal of the study was to review CT examinations of the heads of rabbits and guinea pigs to identify and classify incidental findings. (2) Methods: 60 CT studies of the heads of rabbits and 65 of guinea pigs presented at 2 Institutions were reviewed and the indications for the study recorded. (3) Results: The presence of CT findings not directly related to the reason for the CT examination was present in 40/60 (66.7%) studies of rabbits and in 42/65 (64.4%) studies of guinea pigs. Most commonly, the incidental findings involved the ears, in 24/60 studies in rabbits and in 29/65 studies in guinea pigs. Incidental findings involved the nasal cavities, respectively, in 9 and 7 rabbits and guinea pigs. Soft tissue mineralization was present in 11 rabbits and 17 guinea pigs. (4) Conclusions: Based on the present study, incidental findings detected on CT studies are often present in rabbits and guinea pigs. Veterinarians should be aware of the possible clinical impact of these findings.

## 1. Introduction

Small mammals, including rabbits and guinea pigs, are often kept as pets, accounting for over 21 million animals in the European Union [1]. Domestic rabbits (*Oryctolagus cuniculus*) are popular household pets, with a recent estimated population of approximately 1 million in the UK [2], while guinea pigs (*Cavia porcellus*) are estimated to account for 1% of the pet population in the UK. 

Both rabbits and guinea pigs are particularly prone to dental disease, with prevalence as high as 90% in rabbits [3] and 30.3% and 23.4% in two previous studies in guinea pigs [4,5]. Computed tomography (CT) is the modality of choice for the investigation of dental disease [6,7], and CT findings of dental diseases in both species are well documented [6,7,8]. Otitis media is another frequently reported disease, with a prevalence of clinical middle ear disease of 57% [9] in rabbits and a much lower prevalence of 0.8% in guinea pigs [4]. Also for the assessment of middle ear disease in rabbits the use of CT is well documented [9]. The ever-more-available use of CT as an advanced diagnostic imaging modality increases the possibility of accidental detection of asymptomatic lesions in different organs [10]. The definition of incidental findings, or “incidentalomas” slightly changed over time and, in diagnostic imaging, refers to an unexpected, asymptomatic lesion discovered by chance during the investigation of an unrelated condition [10,11]. In veterinary medicine, the prevalence of CT-detected incidental findings has been reported in the feline head [12], canine adrenal [13] and thyroid gland [14], and canine sacculiths [15]. Recently, incidental findings in CT examinations of dogs and cats have been reported in 11.13% and 8.16% of the studies of the head, respectively [10]. 

The objectives of the present study were to review medical records and CT examinations of the heads of rabbits and guinea pigs and to investigate findings interpreted as unexpected and not related to the clinical indications for the CT examinations.

## 2. Materials and Methods

This study was conducted in a retrospective, bi-institutional, and descriptive design. The animals were presented at the Vetsuisse Faculty, University of Zurich, Switzerland (Institution 1) or at the Department of Veterinary Sciences, Pisa, Italy (Institution 2) for suspected pathologies of the head between March 2019 and May 2023. Written owner’s consent was obtained for each patient prior to diagnostic work-up. Animals undergoing a CT examination of at least the entire head (rostral teeth up to at least the first cervical vertebra) and with complete medical records (including signalment, age, clinical symptoms, and indications for the study) were included. Patients were excluded if the CT study was not diagnostic and if the medical records were incomplete or not available for review. 

CT studies of the head were acquired at Institution 1 with a 16-slice CT scanner, Brilliance CT, and, after October 2022, with a spiral IQon Spectral (Philips, Zurich, Switzerland), with the following settings: Slice thickness 0.8 mm, 120 kVp, 146 and 130 mA, Spiral Pitch Factor 0.288. At Institution 2, CT studies were acquired with the helical Revolution ACT (GE Medical System, Bergamo, Italy) with the following settings: Slice thickness 1.25 mm, 100 kVp, 120 mA, Spiral Pitch Factor 0.75.

All patients underwent general anesthesia and were positioned in sternal recumbency. Because all animals were clinical patients, the anesthetic protocols were not standardized but selected on a case-by-case basis by the treating clinician. For the post-contrast studies, 2 mL/kg of iodinated non-ionic contrast medium was injected (Institution 1: Accupaque 350, 350 mg of I/mL, GE Healthcare, Glattbrugg, Switzerland; Institution 2: Iopamiro, 300 mg/mL Bracco, Milan, Italy) as a bolus with a programmable injector at Institution 1 (Accutron CT-D Medtron Injector, SMD Medial AG, Traegerwilen, Switzerland, injection rate of 2 mL/s) and manually at Institution 2. The late post-contrast phase was scanned between 30 and 60 s post-injection. 

CT studies were retrieved from the Picture Archiving and Communication System (PACS) and reviewed on a workstation Horos^TM^ (Horosproject.org, Annapolis, MD, USA). Images were reviewed in a dynamically adjustable soft tissue window setting using transverse and multiplanar reconstructions (MPR) for interpretation.

From the medical records, the following information was recorded: the species and breed of the animal, sex, gonadal status, date of birth, body weight, age at the time of the CT examination, indication for the CT examination, regions scanned, and availability of the post-contrast study. The indication for CT examination was classified in one of the following groups: suspected dental pathology, anorexia, soft tissue swelling, nasal discharge, ocular discharge, neurological symptoms, weight loss, apathy, exophthalmos, otitis, others, or a combination of more than one indication. The classification was based on medical records. Following discussion among 3 observers (FDC, CP, and SC), all experienced in small mammal CT interpretation, the CT findings and abnormalities were recorded following the classification reported in the Supplementary Materials (Table S1). All the findings were subjectively graded as mild, moderate, or severe, when applicable.

### Statistical Analysis

Data were coded in Excel version 16.45 (Microsoft^®^, Redmond, WA, USA) and analyzed with SPSS version 27.0 (IBM SPSS Inc., Armonk, NY, USA). Descriptive statistics such as means/medians and standard deviations/ranges were computed as appropriate. A statistical analysis was provided for rabbits and guinea pigs separately. For discrete variables such as gender and breed, the prevalence of findings and relative frequencies were calculated.

## 3. Results

### 3.1. Rabbits

In total, 60 CT examinations of rabbits were reviewed. 55 different rabbits were examined. Four animals were scanned more than once (three animals were scanned twice, one animal three times). Of the patients scanned more than once, one each has been rechecked for dental disease, fracture, and otitis. The patient scanned three times, has been rechecked for dental disease at the latest time point with additional otitis. 19 (34.5%) were dwarf rabbits, 11 (20%) lop rabbits, 3 (5.5%) large breeds, 1 (1.8%) lionheads, and 21 (38.2%) unspecified rabbit breeds. 32/55 (58.2%) of the animals were male (6 (10.9%) intact, 26 (47.3%) neutered), and 23/55 (41.8%) were female (8 (14.5%) intact, 15 (27.3%) neutered). The mean body weight of the rabbits at the time of the CT examination was 1902.75 ± 695.53 g. The mean age at the time of the CT examination was 64.1 ± 24.74 months. Indications for the CT study were reported in Table 1. Of the reviewed examinations, 43 studies included only the head (25 native, 18 with late post-contrast phase), 7 included the head and the thorax (3 native, 4 with late post-contrast phase), and 10 included the head, thorax, and abdomen with late post-contrast phase. CT abnormalities considered incidental with respect to the indication for the study were present in 40/60 (66.7%) studies. In 22/40 (55%) studies, incidental findings involved one organ, in 14/40 (35%) two organs, and in 4/40 (10%) studies three organs. The mean number of organs affected by CT-detected incidental findings was 1.03.

**Figure 1:**
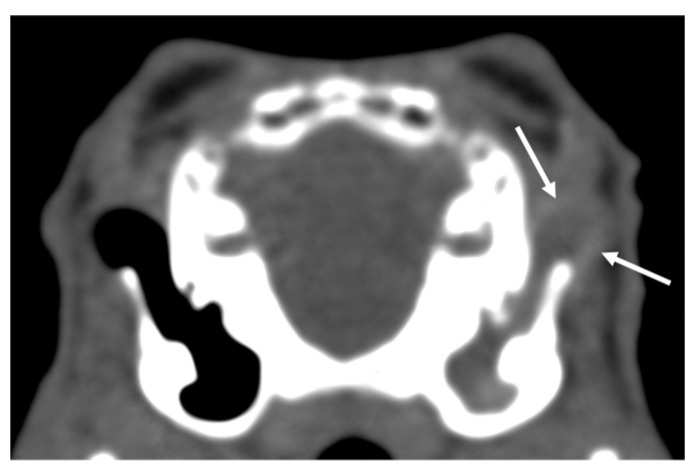
Transverse CT image of the tympanic bullae of one of the rabbits in the study, soft tissue algorithm. Complete soft tissue attenuation of the left external ear canal with a hypoattenuating center and hyperattenuating periphery was interpreted as aural diverticulosis (white arrows). Additional soft tissue attenuation of the bulla tympanica and thickening of the wall were present on the left. This animal was presented with and diagnosed with dental disease. The presence of otitis media and externa was considered incidental. The left side of the animal on the right side of the image.

**Figure 2:**
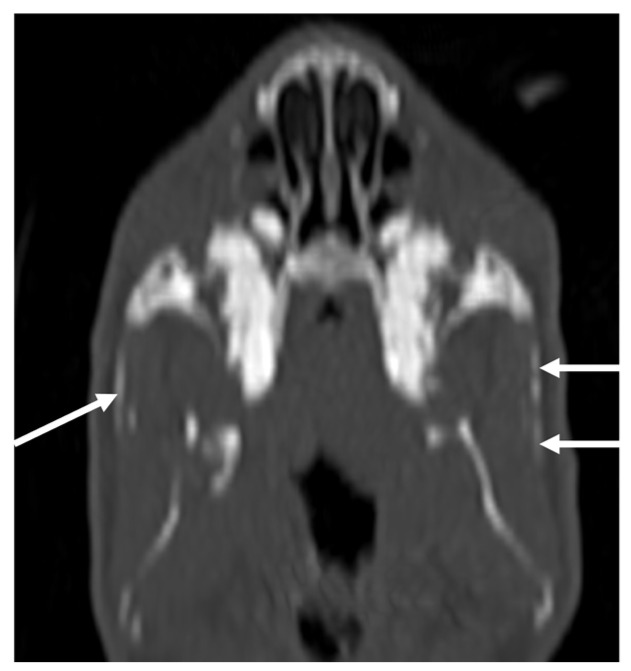
MPR dorsal plane of the CT examination of one of the rabbits in the study, bone algorithm. Linear mineral opacities are visible in the soft tissue of the head (white arrows). This animal was presented with and diagnosed with dental disease. The left side of the animal on the right side of the image.

The most common incidental finding was an ear abnormality. CT findings in the ears were present in 24 studies. In five studies, ear pathology was unilateral. In 11 bullae tympanicae, there was increased attenuation, graded as mild and moderate each in 1 ear and severe in 9. Thickening of the bulla wall was present in six bullae, always graded as mild. In two cases, lysis of the bulla was present, one graded as mild and one as severe. In 32 ears, increased attenuation was present in the external ear canal. In nine of these cases, the material was hypoattenuating with a hyperattenuating rim, interpreted as aural diverticulosis [16] (Figure 1). In eight studies, findings were present both in the bulla tympanica and in the external ear canal. Incidental CT findings in the nose were present in 9 studies and 11 nasal cavities. In all cases, CT findings were interpreted as rhinitis. The pathology was always graded as mild and, in only one cavity, as moderate with loss of conchae. Concurrent involvement of the nasolacrimal duct was present in two animals, in one of them bilaterally with one lysis of the wall. In two studies, there was increased attenuation of the retrobulbar space; in another case, there was a space-occupying lesion associated with the exophthalmus. No abnormalities in the temporomandibular joints were detected. In 11 animals, linear mineralization of the soft tissues of the head and neck was present (Figure 2). In one animal, mild and focal soft tissue swelling was present at the level of the cheek and not associated with other CT-visible lesions. In six studies, dental pathologies were considered incidental and always graded as a minor lesion (Table S1). Of the 17 CT examinations of the thorax, 13 were interpreted as normal. Diagnosed lesions were thymoma, mild lung infiltrate, lung nodules, and lymphadenomegaly, one each in different patients. Of the 10 CT examinations of the abdomen, two were interpreted as normal. Diagnosed lesions were: ovarian and uterine cysts, lipoma, space-occupying lesions in the subcutis, metrophathy (in 2 patients), kidney and liver mineralization, and an abnormally filled intestinal tract.

### 3.2. Guinea Pigs 

In total, 65 CT examinations of guinea pigs were reviewed. 61 different guinea pigs were examined. Four animals were scanned twice. Of the patients scanned more than once, one each had a recheck for otitis and for dental disease. Two patients have different symptoms at the two time points of CT examinations (one with dental disease and later neurological symptoms, one with nasal discharge and later anorexia). 33/61 (54.1%) animals were male (3/61 (4.9%) intact, 30/61 (49.2%) neutered), 28/61 (45.9%) were female (23/61 (37.7%) intact, and 5/61 (8.2%) neutered). The mean body weight at the time of the CT examination was 887.34 ± 195.28 g. The mean age at the time of the CT examination was 38.5 ± 21.02 months. Indications for the CT study were reported in Table 1. Of the reviewed examinations, 45 studies included only the head (32 native, 13 with late post-contrast phase), 11 included the head and the thorax (7 native, 4 with late post-contrast phase), and 9 included the head, thorax, and abdomen (4 native, 5 with late post-contrast phase). CT abnormalities considered incidental with respect to the indication for the study were present in 42/65 (64.6%) examinations. In 22/42 (52.3%) studies, the incidental finding involved only one organ. Incidental findings involved 2 organs in 14/42 (33.3%) studies, 3 organs in 4/42 (9.5%) studies, and 4 or more organs in 2/42 (4.8%) studies. The mean number of organs affected by CT-detected incidental findings was 1.69. 

**Figure 3:**
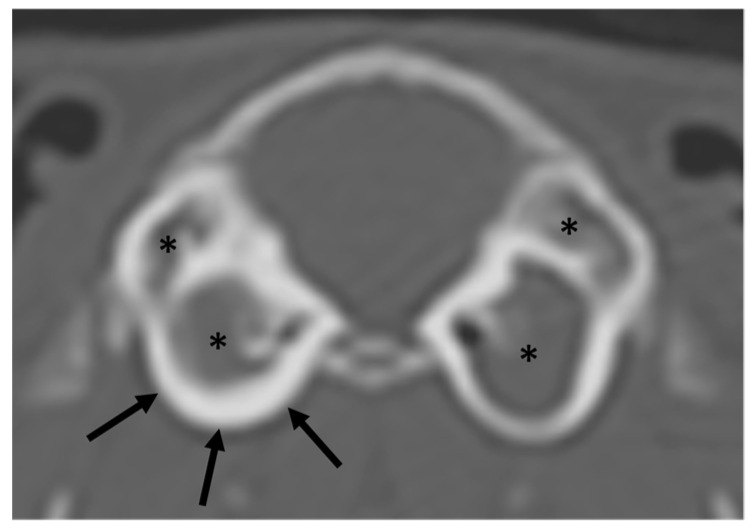
Transverse CT image of the tympanic bullae of one of the guinea pigs in the study, bone algorithm. Bilateral complete filling of the bullae tympanicae with soft attenuating material graded as severe (asterisks). Concurrent thickening of the wall of the bullae tympanicae right (black arrows), graded as moderate. This animal was presented with and diagnosed with dental disease. The presence of otitis media was considered incidental. Left side of the animal on the right side of the image.

**Figure 4:**
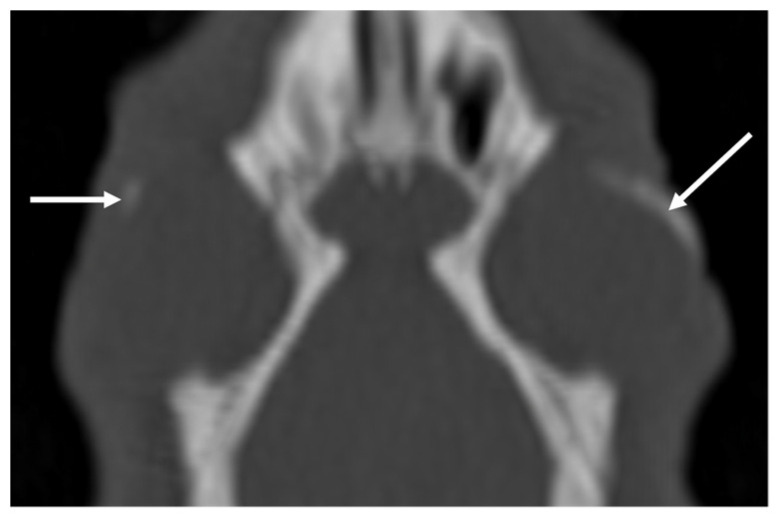
MPR coronal reconstruction of the CT exanimation of one of the guinea pigs of the study, bone algorithm. Example of the ocular mineralization (white arrows), interpreted as heterotopic bone formation (HBF). In this case, it was graded as moderate on the left and mild on the right. This animal was presented and diagnosed with nasal disease. Left side of the animal on the right side of the image.

The most common incidental finding was an ear abnormality. CT findings in the ears were present in 29 studies. In 11 studies, ear pathology was unilateral. In 34 bullae tympanicae there was increased attenuation. In 17, the increased attenuation was present both in the ventral and in the dorsal bullae (Figure 3), in 10 ears in the ventral only, and in 7 ears in the dorsal only. The increased attenuation was graded as mild in 20 ears, moderate in 6, and severe in 6. The thickening of the bulla wall was present in 16 bullae, graded as mild in 11 ears, moderate in 3 ears, and severe in 2 ears. In no case, lysis of the bulla was present. In one ear, increased attenuation was present in the external ear canal, unilateral, right, and mild. In 10 ears, more than one finding was present (increased attenuation and wall thickening). In 10 nasal cavities (and in 7 studies), there was increased attenuation, in 3 of them bilaterally. In all cases, it was graded as mild. The only CT abnormality detected in the nasolacrimal duct was the presence of gas inclusions in 3 animals, in one bilaterally and in the other 2 animals unilaterally (one on the right and one on the left). In 26 eyes (and 17 studies), mineralization was present. In 9 studies, the mineralization was bilateral. With the exception of one animal, graded moderate, the mineralization was graded mild (Figure 4). Deformation of the temporomandibular joints was visible in 3 animals, 2 of them bilateral. In four studies, dental disease was considered incidental and always graded as a minor lesion (Table S1). One guinea pig has an unexpected cavernous space-occupying lesion and one has a focal soft tissue swelling not associated with dental disease. Of the 20 CT studies of the thorax, 4 were interpreted as normal. Diagnosed lesions were lung infiltrate (in 11 animals graded as mild and in 5 as moderate) and a chronic fractured vertebra. Of the nine CT studies of the abdomen, two were interpreted as normal. Diagnosed lesions were: ovarian cysts (in 5 patients), metropathy, adrenomegaly, uroliths (in 2 patients), kidney mineralization, hepatopathy, osteoarthritis of the shoulders, lumbosacral degenerative lesions, and abdominal effusion. 

## 4. Discussion

Incidental findings are unrelated to the chief complaint and not pertinent to immediate patient care [17]. The present study describes the high number of incidental findings detected during the CT examinations of the heads of rabbits and guinea pigs in two Institutions. In 2/3 of the studies in rabbits (40/60, 66.7%) and almost 2/3 in guinea pigs (42/64, 64.6%), incidental findings were detected (Table 2). The mean number of organs involved in incidental findings was more than 1 in both species. Interestingly, both the indications for CT examination and the incidental findings are substantially overlapping in these species. 

In both species, the most common pathology considered an incidental finding was ear disease. In rabbits, that is not surprising, as subclinical middle ear disease and the corresponding CT features are widely documented [9,18]. Also in guinea pigs, otitis media mostly follows a subclinical course [4], but the incidence is not known. It has been reported that thickening of the bulla tympanica alone is not necessarily associated with confirmed inflammation [4]. The lack of soft tissue attenuation in bullae tympanicae with thickened walls could confirm this data. The clinical signs of ear disease, depending on the distribution and severity of the pathology, can be very variable, ranging from scratching and swelling to altered ear position and some neurological signs [9,18]. In the presence of CT findings attributable to ear disease but a lack of clinical signs, the ear pathology is considered subclinical [12]. According to this definition, in the analyzed population, the overall prevalence of subclinical ear disease was mildly lower as reported in rabbits, at 40% [9] and higher, at 44.6% in guinea pigs. Differently compared to rabbits, guinea pigs have a small dorsal and a large ventral bulla tympanica [19]. In half of the analyzed population, both bullae have increased attenuation, suggesting that when an abnormality is present, it often affects both bullae at the same time. If only one bulla is affected, it is more often the ventral one (10/17 cases). Lesions of the wall of the bulla tympanica, a finding that suggests chronicity of pathology, were present only in 2 rabbits with mild lysis (grade III following the published grading [18]) and in 2 guinea pigs with severe thickening. The possible progression of the disease cannot be predicted with the data of our study because of the very small number of re-check examinations and consequently, the clinical relevance of these findings remains unknown. In both species, the prevalence of incidental findings in the middle ear was higher than reported in cats [12]. 

The external ear canal is commonly affected in rabbits with 32 ear canals with increased attenuation. Of them, nine were interpreted as aural diverticulosis [16]. In contrast to rabbits, in guinea pigs, mild increased attenuation of the external ear has been visible in only one ear, suggesting a lower incidence of otitis externa in this species. 

Respiratory diseases are common in rabbits, often secondary to dental pathologies because of the close anatomic relationship. CT of normal and affected nasal cavities has been described [20]. In the analyzed rabbit population, 11 nasal cavities were considered to have increased attenuation and were interpreted as rhinitis or fluid accumulation, not secondary to visible dental pathologies. In only two nasal cavities, it was graded as severe. Rhinitis has been reported in 1% of the guinea pigs in a retrospective study [4]. Similarly to rabbits, 10 nasal cavities of the analyzed guinea pigs had CT findings of increased attenuation. In all cases, it has been graded as mild. The nasal cycle has been described in healthy dogs with CT characteristics previously reported in inflammatory diseases [21]. If a similar process is also present in small mammals, the interpretation of the CT finding as rhinitis could lead to an overestimation of the disease. 

In both species, soft tissue mineralization has been detected in many studies, with a clearly different distribution between the two species. In rabbits, linear mineralization was present in the muscular tissues of the head in 11 animals. Soft tissue mineralization has been reported in rabbits fed a diet containing excess vitamin D [22] and in rabbits with renal pathologies [23,24]. Often, this mineralization affects the walls of blood vessels. In the present study, none of the rabbits had a record of renal insufficiency. In three of the rabbits with mineralization, a post-contrast study was performed, and the mineralization was not macroscopically attributable to blood vessel walls. It is possible, however, that this mineralization involved the walls of smaller vessels or arteries. The rabbits with soft tissue mineralization have a mean age of 79.7 months, slightly higher than the overall mean age of the analyzed population (approximately 64 months). It can be speculated that soft tissue mineralization tends to occur more often in older animals or that these rabbits could suffer subclinical nephropathy. Unfortunately, no information about the diet of the animals was available. In guinea pigs, the mineralization of the soft tissues were visible exclusively in the eyes as pinpoint or short linear mineral attenuating structures in the wall, mainly in the region of the ciliary bodies. The prevalence of this finding was 26%. In this species, heterotopic bone formation (HBF) has been reported [25,26,27], probably occurring as a result of vitamin C secretion into the aqueous. This condition, mainly diagnosed based on clinical appearance and often not related to ocular disfunction [27], has been reported with a much lower prevalence (of 0.8%) with ophthalmic examination [27]. It could be speculated that the detection of HBF could increase with CT examination. The mean age of animals with presumed HBF was 40.65 months, similar to the mean age of the overall analyzed population (38.5 months). In one animal with HBF (bilateral and graded as mild), a retrobulbar space-occupying lesion considered secondary to pathology of the upper cheek teeth and matching the clinical signs of the animal was diagnosed. In this animal, HBF has been considered an independent and incidental finding. 

No abnormalities of the temporomandibular joints were detected in any rabbit, and only mild deformations were visible in three guinea pigs, all with flattening of the mandibular condyle. None of these animals has clinical signs or other CT findings possibly correlating with the pathology of the joint. One possible explanation is that these changes in morphology could represent anatomical variation. 

In two studies of rabbits, there was an incidental increase in the attenuation of the retrobulbar space. Possible differential diagnoses are inflammation or infections of the retrobulbar soft tissues not associated with dental disease. In both animals, the indication was suspicious of otitis, confirmed by CT. In another rabbit, there was a space-occupying lesion associated with mild exophthalmos, considered an incidental finding. In this animal, the exophthalmos was not detected clinically, the indication for the CT study was dental disease (confirmed in the mandible), and the space-occupying lesion was not associated with visible dental disease.

Dental pathologies considered incidental were present in 10 animals: 6 rabbits and 4 guinea pigs, and were always classified as minor lesions (Table S1). Two rabbits were presented with the unspecific sign of apathy. It is impossible to prove if the minor dental changes could have been responsible for the symptomatology, considering that no other lesions were visible in the CT examination. In these cases, the classification as incidental findings followed the review of the medical records and was based on additional diagnosed pathology or on resolution of the clinical signs with no intervention on the teeth. In three guinea pigs, the clinical complaint was soft tissue swelling, and the animals were diagnosed with space-occupying lesions not associated with visible dental pathology. The other guinea pig presented with neurological signs and was diagnosed with otitis media. 

Overall, incidental findings detected in our study (in 66.7% and 64.6%, respectively, in rabbits and guinea pigs) are much higher than described in dogs and cats, with the presence of incidentalomas in respectively 11.65 and 8.33% of the CT examination of the head [10].

The major limitation of the study is the impossibility of proving that the defined incidental findings have really had no influence on the clinical symptoms of the patients. The conclusion we drew to classify a finding as incidental follows the medical records and reflects the ultimately partially subjective assessment of patients by the treating clinician. The clinical complaint—and secondary indication for the CT examinations—was not always specific. Three rabbits were presented with anorexia and three with apathy. In five of these animals, dental pathologies were present, and they were considered a possible explanation for the clinical symptoms. In one animal with anorexia, the only finding was mild rhinitis. In this case, the link between symptomatology and CT findings remains unknown. Seven guinea pigs were presented with anorexia (one of them with weight loss) and one with weight loss only. In six of them, dental pathologies were present and considered a possible explanation for the clinical signs. In only one animal, presented with anorexia, the only finding was HBF, considered incidental. The relatively high number of CT examinations of additional anatomical regions (thorax and abdomen) reflects the difficulty of a precise suspected diagnosis. The retrospective and descriptive nature of the study is also a limitation, causing selection bias for the retrospectively available subpopulation and not allowing independent randomization. The classification of the indications for the CT examination was based on medical records and impossible to standardize. Some minor clinical symptoms may have been undetected or overlooked by the owners and the treating clinician and then become virtually invisible from the retrospective analysis of medical records. Typically, the patients were not under current treatment at the time of the diagnostic workup, because they still lacked a definitive diagnosis. Exceptions are obviously patients undergoing a follow-up CT examination. It cannot be excluded, however, that some patients received some previous treatments not reported on medical records. Moreover, the time frame between the presence of symptoms and the CT examination was not investigated, and it could play a role in the ability to detect a lesion. The difference in CT equipment can also be considered a limitation of the study, as is typical of multicentric and clinical studies. A CT scan with different technical parameters might result in different sensitivity in finding recognition and diagnostic ability. The lack of a post-contrast phase in every CT examination has also to be considered. The reason for performing or not performing a contrast study of the head in addition to the native examination was not always reported in the clinical records and was not standardized. It can be speculated that the availability of a post-contrast study may be secondary to the presence of venous access, owner compliance, economic reasons, the preference of the clinician, and the perceived need for the diagnosis of the suspected disease. For the evaluation of only dental disease, contrast studies may not be considered essential. The described incidental findings, regarding most often the nose and ears, were best evident in native bone algorithm studies. The cavernous characteristics of soft tissue lesions are often recognizable in the native study. However, it cannot be excluded that post-contrast studies may have yielded additional information able to change the number of detected lesions and/or their classification. 

The choice to perform a CT examination as a diagnostic workup was also not standardized. Typically, the CT examination is the first choice in animals suffering from presumed dental disease because of its known diagnostic superiority [28], as well as in animals presented with suspected pathology of the head (exophthalmos, otitis, space-occupying lesions, neurological symptoms). In patients with nonspecific symptoms only (like weight loss, apathy, or other symptoms), the CT examination was typically performed after an inconclusive radiographic study of the thorax and abdomen or abdominal ultrasound. The reported prevalence of incidental findings, moreover, refers to the CT examinations. Considering that a small number of animals were scanned more than once, a lower prevalence has to be expected considering the patient population. The examined population consisted of clinical patients and elderly animals, with a mean age at the time of the CT examination of 64.1 ± 24.74 months in rabbits and 38.5 ± 21.02 months in guinea pigs. That could explain the high number of reported incidental findings, likely due to the increasing prevalence of comorbidities and degenerative diseases in older animals. The clinical relevance of the recorded incidental findings has not been investigated as well, and it remains unknown if and which of these findings will become relevant in the future. Lastly, the diagnosis is based on the CT findings, and considering the findings were incidental, often no further diagnosis (such as cytology or histopathology examination) was pursued.

## 5. Conclusions

In conclusion, the present study describes that incidental findings are often detected in CT examinations of the heads of rabbits and guinea pigs in approximately 2/3 of the analyzed population. This will increase awareness of the lesion’s recognition and potential need for clinical attention. Further studies are necessary to investigate the need for clinical intervention or follow-up for lesions that are potentially clinically relevant for the patient.

## Figures and Tables

**Table 1:** Indications for the CT studies in rabbits and guinea pigs. In each species, in the left column, a single indication for the CT examination is reported. In the right column, the additional indication(s) for the CT examination are reported. In brackets, the number of CT studies for each category.

Rabbits (60)		Guinea Pigs (65)	
Only indication for the CT examination (n)	Additional indications for the CT examination (n)	Only indication for the CT examination (n)	Additional indications for the CT examination (n)
Dental pathology (16)	Otitis (3) Swelling (2) Nasal discharge (1) Nasal discharge and otitis (1) Anorexia (1)	Dental pathology (16)	Swelling (6) Anorexia (2) Weight loss (1) Neurological symptoms and anorexia (1)
Anorexia (3)	Ocular discharge (1)	Anorexia (6)	Swelling (2) Weight loss (1)
Swelling (9)	Nasal discharge (1)	Swelling (17)	
Nasal discharge (5)	Otitis (1) Ocular discharge (1)	Nasal discharge (2)	Weight loss (1)
Otitis (3)	Nasal discharge (1)	Otitis (2)	
Neurological symptoms (3)		Neurological symptoms (3)	
Exophthalmos (2)		Exophthalmos (2)	
Ocular discharge (1)		Weight loss (1)	
Others	Apathy (3) Fractured mandibula (1) Blood in endotracheal tube (1)	Others:	Stridor (1) Swallowing problems (1)

**Table 2:** Summary of CT findings considered incidental in rabbits (in light) and guinea pigs (in bold). The number of the affected organs are reported in brackets. In the same animal and in the same organ, one or more findings could have been present.

Anatomical Region	CT Abnormalities Detected			
Nasal cavities	Increased attenuation/rhinitis (11/**10**)	Space-occupying lesion (0/**0**)	Loss of conchae (1/**0**)	
Nasolacrimal duct	Enlargement (3/**0**)	Wall thickening (0/**0**)	Wall lysis (1/**0**)	Presence of gas (0/**4**)
Bulla tympanica	Increased attenuation (11/**34**)	Enlargement (0/**0**)	Thickening of the wall (6/**16**)	Lysis or irregularities of the wall (2/**0**)
Internal ear	Deformation (0/**0**)	Lysis (0/**0**)		
External ear	Increased attenuation (23/**1**)	Displacement (0/**0**)	Aural diverticulosis (9/**0**)	
Retrobulbar space	Increased attenuation, loss of fat tissue (2/**0**)	Space-occupying lesion (1/**0**)		
Eye	Exophthalmos (1/**0**)	Deformation (0/**0**)	Lens luxation (0/**0**)	Others: mineralization (0/**26**)
Maxilla/mandibula	Minor lesions (6/**4**)	Major lesions associated with tooth (0/**0**)	Aggressive bone lesions not associated with tooth (0/**0**)	Fracture (1/**0**)
TMJ *	Luxation (0/**0**)	Fracture (0/**0**)	Deformation (0/**5**)	Lysis (0/**0**)
Soft tissues	Diffuse swelling (1/**1**)	Solid space occupying lesion (0/**0**)	Cavernous space occupying lesion (0/**1**)	Mineralization (11/**0**)

* Temporomandibular joint.

## Data Availability

The data not presented in the manuscript are available for consultation after a reasonable request to the corresponding authors.

## Supplementary Materials

The following supporting information can be downloaded at: https://www.mdpi.com/article/10.3390/vetsci10080504/s1, Table S1: Classification and description of the CT findings.
