# Supplementary material for: Incidental Findings in Computed Tomography Examination of the Head in Rabbits and Guinea Pigs
Source: Vet Sci. 2023 Aug 4;10(8):504. doi: 10.3390/vetsci10080504 (PMC10459266; doi:10.3390/vetsci10080504)
Supplement: Supplementary file 1 [file vetsci-10-00504-s001.zip › vetsci-2503571-Supplementary Material.pdf]

veterinary sciences

MDPI

Table S1. Description and classification of the CT finding of the CT studies of the head in rabbits and guinea pigs.

Organ	Lesion description			
Nasal cavities	Increased attenuation (rhinitis)	Space-occupying lesion	Loss of conchae	
Nasolacrimal duct	Enlargement	Wall thickening	Wall lysis	Displacement
Bulla tympanica	Increased attenuation	Enlargement	Thickening of the wall	Lysis or irregularities of the wall
Internal ear	Deformation	Lysis		
External ear	Homogeneous increased attenuation	Displacement	Heterogeneous increased attenuation, mass effect (aural diverticulosis)	
Retrobulbar space	Increased attenuation/loss of fat tissue	Space-occupying lesion		
Eye	Exophthalmos	Deformation	Lens luxation	Rupture
Maxilla/ Mandibula	Minor lesions (bulging around tooth root, with or without focal lysis)	Major lesions (aggressive bone lesions) associated with tooth	Aggressive bone lesions not associated with tooth	Fracture
TMJ*	Luxation	Fracture	Deformation	Lysis
Soft tissues	Diffuse swelling	Solid space occupying lesion	Cavernous space occupying lesion	

* temporomandibular joint.
